# Regulation of Src family kinases by muscarinic acetylcholine receptors in heterologous cells and neurons

**DOI:** 10.3389/fnmol.2023.1340725

**Published:** 2024-01-11

**Authors:** Li-Min Mao, Lexi Young, Xiang-Ping Chu, John Q. Wang

**Affiliations:** ^1^Department of Biomedical Sciences, School of Medicine, University of Missouri-Kansas City, Kansas City, MO, United States; ^2^Department of Anesthesiology, School of Medicine, University of Missouri-Kansas City, Kansas City, MO, United States

**Keywords:** Src, Fyn, tyrosine kinase, M_1_ receptor, M_4_ receptor, striatum

## Abstract

Five muscarinic acetylcholine (mACh) receptor subtypes are divided into two classes: the M1 class (M_1_, M_3_, and M_5_) and the M2 class (M_2_ and M_4_). The former is coupled to G_q_ proteins, while the latter is coupled to G_i/o_ proteins. Accumulating evidence indicates that mACh receptors play a significant role in the regulation of the Src family kinase (SFK), a subfamily of non-receptor tyrosine kinases. mACh receptors exert their roles in a subtype-dependent fashion and preferentially target Src and Fyn, two members of SFKs that are expressed in the brain and enriched at synaptic sites. While the M_1_ receptor positively modulates SFK activity, the M_4_ receptor inhibits it. By modulating SFKs, mACh receptors are actively involved in the regulation of expression and function of a variety of receptors, structural proteins, and signaling molecules. In particular, the M_4_ receptor and the dopamine D_1_ receptor are coexpressed in striatonigral projection neurons of the striatum. G_i/o_-coupled M_4_ and G_q_-coupled D_1_ receptors antagonistically regulate SFK activity, thereby forming a dynamic balance controlling glutamate receptor activity, excitability of neurons, and synaptic plasticity. In summary, mACh receptors play a crucial role in regulating SFK activity in heterologous cells and neurons.

## Introduction

The non-receptor tyrosine kinase (nRTK) family consists of a panel of kinases that tyrosine-phosphorylate proteins and thereby regulate a variety of cellular and signaling activities involving the phosphorylation-modified proteins. In the central nervous system, nRTKs play a pivotal role in the regulation of neuronal and synaptic activities and in the pathogenesis and symptomatology of various neurological and neuropsychiatric disorders (Neet and Hunter, 1996). The Src family kinase (SFK) is a subfamily of nRTKs. Several SFK members (Src, Fyn, Yes, Lyn, and Lck) are expressed in the brain (Omri et al., 1996; Kalia et al., 2004; Bongiorno-Borbone et al., 2005). Noticeably, Src and Fyn are enriched at synaptic sites (Ohnishi et al., 2011). In addition to their abundant postsynaptic presence, Src and Fyn reside and function presynaptically (Onofri et al., 1997; Nakamura et al., 2001). As such, both SFK members are implicated in the modulation of synaptic transmission and plasticity (Kalia et al., 2004; Ohnishi et al., 2011; Schenone et al., 2011). A large number of substrates of Src and/or Fyn have been identified in the cytoplasmic and synaptic compartments, including receptors, ion channels, enzymes, signaling molecules, etc. By binding to these substrates, SFKs phosphorylate specific tyrosine sites on them and dynamically modulate their expression and function.

Acetylcholine is an essential neurotransmitter in the mammalian brain. This transmitter interacts with nicotinic and muscarinic acetylcholine (mACh) receptors to achieve its action. While nicotinic receptors are ion channels, mACh receptors are G protein-coupled receptors (GPCR). Based on the type of G proteins that mACh receptors are connected to, five subtypes of mACh receptors (M_1_–M_5_) are divided into two classes: the M1 class and the M2 class (Caulfield and Birdsall, 1998). The M1 class of mACh receptors includes M_1_, M_3_, and M_5_ subtypes which are coupled to G_q_ proteins. The M2 class, i.e., M_2_ and M_4_ subtypes, is coupled to G_i/o_ proteins (Wess, 1996). As such, activation of the M1 class activates phospholipase Cβ1 (PLCβ1), yielding two downstream signaling molecules, diacylglycerol (DAG) and inositol-1,4,5-triphosphate (IP_3_). The former activates protein kinase C (PKC), whereas the latter induces Ca^2+^ release from the intracellular Ca^2+^ stores. Activation of the M2 class inhibits adenylyl cyclase, leading to the reduction of cAMP formation and inhibition of protein kinase A (PKA) activity. By triggering distinct signaling pathways, mACh receptors exert the receptor subtype-specific regulation of neuronal and synaptic activities.

SFK activity is regulated by changing cellular and synaptic input in a phosphorylation-dependent manner. The upregulated SFK activity is seen following an increase in autophosphorylation at a specific site. That is, phosphorylation of SFKs at a conserved residue, pan tyrosine 416 (Y416), within the activation loop results in activation of SFKs (Roskoski, 2005; Okada, 2012). Multiple neurotransmitters have been found to regulate SFKs through altering Y416 phosphorylation. Among these transmitters is acetylcholine. Accumulating evidence shows that stimulation of mACh receptors has a profound impact on SFK activity in transfected mammalian cells and in neurons at various brain regions. For instance, pharmacological stimulation of the M1 class of mACh receptors activates SFKs and thereby regulates a discrete set of downstream targets. Meanwhile, stimulation of the M2 class, especially the M_4_ subtype, has a significant impact on SFK activity, leading to changes in SFK-mediated tyrosine phosphorylation of a number of substrates. We in this review summarize the mACh receptor-mediated regulation of SFKs in heterologous cells and neurons. Of note, mACh receptors and SFKs are broadly distributed in the mammalian brain. Their expression and interactions in the hippocampus may play significant roles in memory, emotion, and cognitive function, while an active state of mACh-SFK coupling in the striatum may be implicated in the modulation of motivation, reinforcement, and reward perception.

## Regulation of SFKs by mACh receptors

GPCRs are linked to SFKs (Berndt and Liebschcer, 2021; Perez et al., 2022) and among these GPCRs is the mACh receptor. A large number of early studies have observed consistent results, establishing that mACh receptors can activate SFKs to regulate a variety of downstream targets. For instance, application of the mACh selective agonist muscarine potentiated *N*-methyl-D-aspartate (NMDA)-evoked currents in acutely isolated hippocampal CA1 pyramidal neurons (Lu et al., 1999; Tian et al., 2016). This muscarine-induced potentiation was blocked by the tyrosine kinase inhibitor lavendustin A, but not its inactive analog lavendustin B. The effect of muscarine was also blocked by a Src inhibitory peptide Src (40–58) but not the scrambled sequence control sSrc (40–58). Since Src (40–58) disrupts the Src interaction with NMDA receptor GluN2A subunits (Gingrich et al., 2004) and thereby selectively blocks the effect of Src but not Fyn on their substrates (Yang et al., 2012), Src was believed to be activated by muscarine to link mACh signals to NMDA receptors. In contrast to Src, Fyn seems insignificant in this event since a Fyn interfering peptide Fyn39–57 did not block the muscarine-induced NMDA current potentiation in hippocampal CA1 neurons (Tian et al., 2016).

In addition to the Src-NMDA receptor pathway (Rajani et al., 2021), mACh receptors have been found to engage SFKs to activate other proteins and signaling pathways. Pharmacological stimulation of mACh receptors with a non-selective agonist carbachol increased phosphorylation of extracellular signal-regulated kinases (ERK) in cultured cortical neurons (Rosenblum et al., 2000). The SFK inhibitor PP1 reduced this increase. Since carbachol retained its ability to activate ERK in cortical cultures from Fyn knockout mice, Fyn may not participate in processing the mACh regulation of ERK. Additionally, carbachol or muscarine stimulated (1) phosphorylation of PKCδ (Benes and Soltoff, 2001) and focal adhesion kinase (FAK) (Jope et al., 1999; Watcharasit et al., 2001), (2) expression of the activity-regulated cytoskeleton-associated gene (ARC) (Teber et al., 2004), (3) secretion of a soluble amyloid precursor protein in human neuroblastoma SH-SY5Y cells (Canet-Aviles et al., 2002), and (4) activity of ERK and/or the cAMP-responsive element-binding protein in rat neural precursor cells (Zhao et al., 2003), MCF-7 human breast cancer cells (Jimenez and Montiel, 2005), and oligodendrocytes progenitors (Cui et al., 2006). All of these carbachol- or muscarine-stimulated events were blocked by PP1 or another SFK inhibitor PP2, indicating that SFKs take part in forming a signaling pathway linking mACh receptors to these targets. In addition to carbachol and muscarine, donepezil (a selective acetylcholinesterase inhibitor) was used in exploring the cholinergic receptor-SFK coupling. Donepezil by inhibiting cholinesterase-catalyzed hydrolysis of acetylcholine increases acetylcholine concentrations at cholinergic synapses. Recent studies demonstrated that donepezil promoted stroke-induced neurogenesis in the rat and mouse subventricular zone (Wang et al., 2017; Man et al., 2020), while the mACh receptor antagonist atropine reduced it in the mouse subventricular zone (Wang et al., 2017). Since the effect of donepezil was abolished by the Src inhibitor KX-01 (Man et al., 2020), active Src is required for linking mACh receptors to enhanced neurogenesis.

Along with the above indirect data observed with SFK inhibitors, evidence for activation of SFKs in response to mACh receptor agonists was obtained by measuring SFK phosphorylation at Y416. Carbachol, for instance, increased SFK phosphorylation at Y416, i.e., activation of SFKs, in pyramidal neurons of rat prefrontal cortex (PFC) slices (Ma et al., 2003) and cultured rat neural precursor cells (Zhao et al., 2003). Activated Src may mediate the carbachol-induced potentiation of γ-aminobutyric acid (GABA)_A_ receptor-mediated currents because (1) the Src inhibitory peptide Src (40–58) but not its control sSrc (40–58) prevented the potentiation of GABA_A_ receptors induced by carbachol, and (2) injecting the active enzyme p60-cSrc into PFC neurons mimicked the effect of carbachol (Ma et al., 2003). Of note, carbachol also enhanced tyrosine phosphorylation of immunopurified Fyn but not Lyn from cultured rat oligodendrocyte progenitors (Cui et al., 2006). In pancreatic acinar cells, the SFK member Yes was activated by carbachol as demonstrated by an increase in Yes-Y416 phosphorylation in response to carbachol (Sancho et al., 2012).

While mACh receptor agonists, probably through activating the M1 class (see below), activate SFKs, mACh receptor antagonists also elevate SFK activity in a specific brain region. In a dopamine-innervated brain region, i.e., the striatum where SFKs (Src and Fyn) and M_4_ receptors are abundantly expressed (Levey et al., 1991; Pascoli et al., 2011), the non-subtype-selective mACh receptor antagonist scopolamine after a systemic injection markedly enhanced SFK Y416 phosphorylation in adult rats *in vivo* (Mao et al., 2018). The scopolamine stimulation of Y416 phosphorylation occurred in the two subdivisions of the striatum, the caudate putamen and nucleus accumbens. Another mACh antagonist atropine produced the similar increase in striatal Y416 phosphorylation. These findings indicate that mACh receptors in the striatum inhibit basal phosphorylation of SFK Y416 under normal conditions, probably via a subtype-specific mechanism involving M_4_ receptors (see below). Of note, scopolamine phosphorylated Fyn rather than Src immunopurified from the striatum, indicating a selective effect of scopolamine on Fyn (Mao et al., 2018). Additionally, coadministration of scopolamine and a dopamine D_1_ receptor agonist SFK81297 at their subthreshold doses induced a significant increase in SFK Y416 phosphorylation in the striatum (Mao et al., 2018). This suggests that the mACh receptor-mediated cholinergic transmission and the D_1_-mediated dopaminergic transmission antagonistically interact with each other to form an intrinsic balance within the striatum controlling SFK homeostasis (see below).

## Regulation of SFKs by the M1 class

Application of carbachol enhanced Src autophosphorylation at Y418 (Y416 in chicken Src), an indicator of Src activation, in rat PFC slices (Ma et al., 2003). Given that M_1_ receptor mRNAs were most abundant in PFC pyramidal neurons (Ma et al., 2003), the M_1_ subtype may participate in mediating the effect of carbachol on Src in these neurons. In support of this notion, the M_1_ antagonist pirenzepine blocked the Src-dependent potentiation of GABA_A_ receptors in rat PFC neurons in response to carbachol (Ma et al., 2003). Moreover, carbachol stimulated G_q_-coupled mACh receptors to increase Y416 phosphorylation of immunopurified Src in HEK293 cells (Vazquez et al., 2004).

The M_1_ receptor-mediated upregulation of SFK activity affects several surface-expressed receptor activities. As aforementioned, muscarine potentiated NMDA receptor activity via activating Src in hippocampal CA1 neurons (Lu et al., 1999; Tian et al., 2016). This potentiation was likely mediated by the M1 class of mACh receptors since (1) the hippocampus is enriched with M_1_ receptors (Levey, 1996), (2) the muscarine-induced potentiation of NMDA receptors in this region was mimicked by the M_1_ agonist xanomeline and was blocked by the M_1_ antagonist pirenzepine (Tian et al., 2016), and (3) inhibition of SFKs with PP2 prevented the carbachol-triggered and M_1_-mediated phosphorylation of NMDA receptor subunits (GluN2B) at Y1472 in primary rat cortical cultures (Chen et al., 2016). In addition to NMDA receptors, M_1_ receptors are believed to engage active Src to potentiate GABA_A_ receptor activity in rat PFC pyramidal neurons (Ma et al., 2003). Moreover, M_1_ receptors interacted with fibroblast growth factor receptors (FGFR) to form heteroreceptor complexes in hippocampus cultures, and as a result, M_1_ receptor signals could readily transactivate FGFRs via a Src-dependent manner (Di Liberto et al., 2017).

M_1_-activated SFKs may serve as a key transducer linking M_1_ receptors to many other downstream effectors. For example, mitogen-activated protein kinases (MAPK) form an essential intracellular signaling pathway. Carbachol via stimulating M_1_ receptors activated the MAPK/ERK pathway in COS-7 cells (Igishi and Gutkind, 1998; Rosenblum et al., 2000). In DT40 cells deficient in Lyn, M_1_ receptors failed to stimulate MAPKs, indicating the role of Lyn in linking M_1_ receptors to MAPK (Wan et al., 1996). The M_1_-regulated ERK phosphorylation was also SFK-dependent in SK-N-MC human brain neuroepithelioma cells (Chan et al., 2005). In addition to the MAPK/ERK pathway, carbachol stimulation of M_1_ and/or M_3_ receptors activated SFKs to elevate ARC expression in SH-SY5Y cells (Teber et al., 2004) and in primary rat cortical neurons (Chen et al., 2016). Activation of G_q_-coupled mACh receptors obligated Src to activate transient receptor potential (TRP) channels in HEK293 cells (Vazquez et al., 2004). Stimulation of M_1_ receptors induced endocytosis of TWIK (tandem of P domains in a weak inwardly rectifying K^+^ channel)-related acid-sensitive K^+^ (TASK)1 channels in rat adrenal medullary cells (Matsuoka and Inoue, 2017).

**FIGURE 1:**
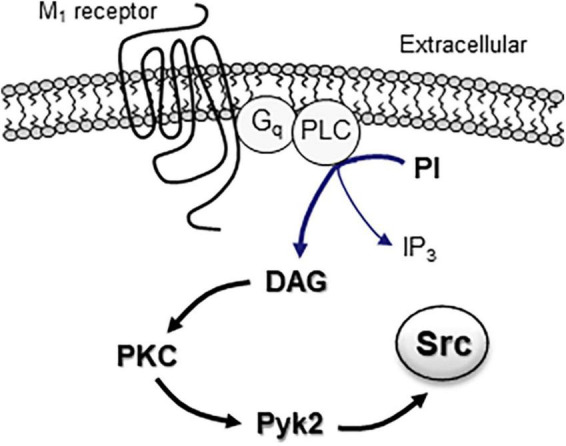
The M_1_ receptor-associated signaling pathway in activation of intracellular Src proteins. Activation of G_q_-coupled M_1_ receptors results in activation of PLC, which subsequently hydrolyzes phosphoinositide (PI) to yield DAG and IP_3_ molecules. DAG functions as an activator of downstream PKC. Active PKC then promotes Src activation through an intermediary protein Pyk2.

The postreceptor signaling pathway(s) linking M_1_ receptors to SFKs have been studied in heterologous cells and neurons. M_1_ receptors are known to be coupled to pertussis toxin-insensitive heterotrimeric G_q_ proteins, including αq, β, and γ subunits. Evidence shows that both G_αq_ and G_β/γ_ dimers play important roles in relaying M_1_ signals to Src. In HEK293 cells, a constitutively active mutant of G_αq_ proteins stimulated SFK activity, and active SFKs then tyrosine-phosphorylated multiple downstream proteins (Nagao et al., 1998). In COS-7 cells stably expressing recombinant M_1_ receptors, stimulating M_1_ receptors induced a PP1-sensitive phosphorylation of MAPKs (Igishi and Gutkind, 1998). Overexpression of G_β/γ_ dimers in these cells also activated MAPKs, which was inhibited by the dominant-negative Src (Igishi and Gutkind, 1998). At the level downstream to G proteins, M_1_ receptors are known to activate PLCβ1 to produce DAG which sequentially activates PKC. After PKC activation, evidence shows that proline-rich tyrosine kinase 2 (Pyk2) (also known as cell adhesion kinase β, CAKβ), a member of FAK family of nRTKs, works as an intermediary protein between PKC and Src (Yang et al., 2014; Figure 1). Indeed, M_1_ receptors were found to activate Pyk2 (Felsch et al., 1998), and active Pyk2 then served as an effector to link PKC to Src in response to M_1_ stimulation in a stable cell line (Felsch et al., 1998) or mACh receptor stimulation in rat PFC slices (Ma et al., 2003). Similarly, in PC12 cells, muscarinic receptor stimulation activated Src through the PKC-Pyk2 pathway, which led to TASK1 channel endocytosis (Matsuoka et al., 2020).

In addition to the M_1_ subtype, the M_3_ subtype appears to be linked to SFKs. In HEK cells stably expressing M_3_ receptors, carbachol showed the ability to activate MAPKs (Slack, 2000). The activation of MAPKs by M_3_ receptor stimulation was partially reduced by PP1, indicating that M_3_ receptors may in part activate PP1-sensitive SFKs to stimulate MAPKs. Similarly, stimulation of M_3_ receptors with the muscarinic receptor agonist pilocarpine caused activation of MAPK/ERK1/2 in mouse insulinoma cells, and the stimulatory effect of pilocarpine was blocked by PP2 (Pronin et al., 2017).

Beyond the MAPK/ERK pathway, M_3_ receptors regulate another downstream kinase via a SKF/Fyn-dependent mechanism. In SH-SY5Y cells expressing M_3_ receptors, the non-subtype-selective mACh agonist oxotremorine-M increased tyrosine phosphorylation of the activated Cdc42Hs-associated kinase-1 (ACK-1), which was blocked by the SFK inhibitor (Linseman et al., 2001). Since loading cells with the Fyn-SH2 or Fyn-SH3 domain that reduced the SH2- and SH3-mediated interactions between Fyn and ACK-1 attenuated the effect of oxotremorine-M, Fyn tyrosine kinase was likely activated by oxotremorine-M to link M_3_ receptors to ACK-1 (Linseman et al., 2001).

## Regulation of SFKs by the M2 class

The M2 class of mACh receptors includes the M_2_ and M_4_ subtypes. Studies conducted in heterologous cell lines *in vitro* reveal the linkage of M_2_ receptors to SFKs. Pharmacological stimulation of M_2_ receptors increased Src but not Fyn activity in cultured colonic smooth muscle cells (Singer et al., 2002) and activated PP2-sensitive SFKs in gastric smooth muscle cells (Mahavadi et al., 2007). M_2_ receptors also activated Src in COS-7 cells (Igishi and Gutkind, 1998) and Fyn in SH-SY5Y cells (Stirnweiss et al., 2006). The M_2_-mediated upregulation of Src seems to be mediated through a signaling mechanism involving G_β/γ_ (Igishi and Gutkind, 1998; Murthy, 2008). In a recent study, M_2_ receptors with the phosphorylated C-terminal tail were able to interact with G_β_ -arrestin-1, which constitutes a necessary and sufficient step to allosterically activate a downstream effector (Src) by promoting Src autophosphorylation *in vitro* (Pakharukova et al., 2020).

At present, the M_2_-SFK relationship in neurons is less well characterized due to limited studies. One study demonstrated that stimulation of M_2_ receptors induced hyperpolarization of local GABAergic interneurons of the mouse thalamus by recruiting G_βγ_, class-1A phosphatidylinositol-4,5-bisphosphate 3-kinase (PI3K), and c-Src, leading to activation of TASK-1 channels in these interneurons (Leist et al., 2017).

Meanwhile, initial studies investigated the potential role of M_4_ receptors in the regulation of SFKs in neurons from the striatum, one of brain regions known for its highest level of M_4_ receptors and its significance in mood, cognitive, and motor functions (Levey et al., 1991; Hersch et al., 1994; Chapman et al., 2011). Pharmacological blockade of mACh receptors by scopolamine readily increased SFK Y416 phosphorylation in the rat striatum (Mao et al., 2018). The scopolamine effect was likely mediated by blocking the M_4_ subtype as M_4_ receptors are inhibitory in nature (i.e., inhibiting the cAMP/PKA pathway, also see below) and represent a subtype from the M2 class expressed in striatonigral output neurons (Levey et al., 1991; Ince et al., 1997; Santiago and Potter, 2001). These data indicate that there exists a tonic M_4_ receptor-dependent inhibition of SKF activity in striatal neurons. As such, pharmacological blockade of these mACh receptors leads to upregulation of SFK activity in the region. Moreover, the M_4_ inhibition appears to function at a relatively high level under basal conditions, given that exogenous application of a systemically active positive allosteric modulator (PAM) selective for M_4_ receptors (VU0152100) exhibited a minimal impact on spontaneous SFK Y416 phosphorylation in the rat striatum (Mao and Wang, 2015).

**FIGURE 2:**
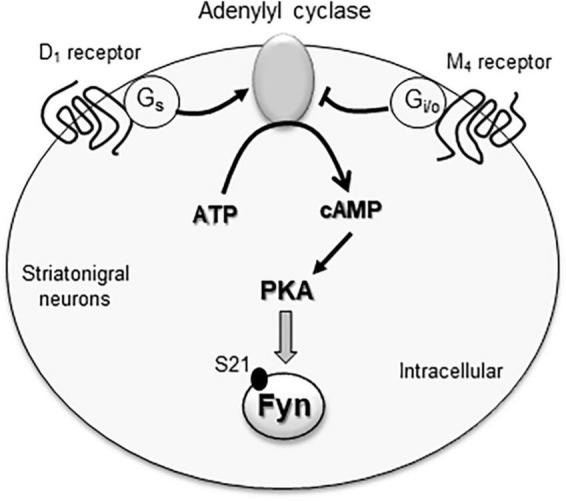
The M_4_ receptor-associated signaling pathway in inhibition of cytoplasmic Fyn. Fyn activity is likely regulated by the adenylyl cyclase/cAMP/PKA pathway. Active adenylyl cyclase is known to increase cAMP formation and thereby activate PKA. Active PKA can then phosphorylate Fyn at a serine site (S21), which is a critical event for maintaining Fyn kinase activity. G_s_-coupled D_1_ receptors and G_i/o_-coupled M_4_ receptors are co-expressed in striatonigral projection neurons within the striatum. Since the two receptors have the opposite effects on adenylyl cyclase, they form a dynamic balance controlling the cAMP/PKA pathway and thus Fyn activity in these neurons.

The postreceptor signaling pathway linking M_4_ receptors to SFK/Fyn may involve cAMP and PKA. As a G_i/o_-coupled receptor, the M_4_ receptor inhibits cAMP formation and thereby reduces PKA activity (Wess, 1996). Interestingly, Fyn contains a PKA recognition motif (RxxS) on its amino terminal SH4 domain. Within this motif, the S21 residue was phosphorylated by PKA, and S21A mutation (phosphorylation-deficient mutation) blocked PKA phosphorylation of Fyn (Yeo et al., 2011). The PKA-catalyzed Fyn S21 phosphorylation was critical for regulating Fyn activity as S21A mutation caused a deficit of the ability of Fyn in modulating cell mobility (Yeo et al., 2011). Thus, M_4_ receptors likely inhibit the cAMP-PKA pathway to suppress Fyn activity (Figure 2). Consistent with this notion, stimulating PKA with forskolin upregulated Fyn activity although not Src activity in spinal neurons (Yang et al., 2011).

In addition to M_4_ receptors, dopamine D_1_ and D_2_ receptors are expressed in the striatum. As the major dopamine receptor subtypes in this region, these two receptors are segregated into two subpopulations of medium spiny projection neurons: D_1_ receptors in striatonigral neuron and D_2_ receptors in striatopallidal neurons (Gerfen et al., 1990; Aubert et al., 2000; Bertran-Gonzalez et al., 2010). Consistent data show that D_1_ receptors stimulate Fyn in the striatum, while D_2_ receptors inhibit it. Specifically, D_1_ agonists and D_2_ antagonists enhanced Fyn although not Src phosphorylation in striatal neurons (Dunah et al., 2004; Hattori et al., 2006; Pascoli et al., 2011; Mao and Wang, 2015, 2016a) as well as hippocampal neurons (Yang et al., 2012). Remarkably, D_1_ and M_4_ receptors are coexpressed in striatonigral neurons (Ince et al., 1997; Santiago and Potter, 2001). This provides a basis for two receptors to crosstalk. It is possible that G_s_-coupled D_1_ receptors and G_i/o_-coupled M_4_ receptors work in concert to form a dynamic balance to regulate the cAMP-PKA pathway and thus Fyn in striatonigral neurons (Figure 2). Consistent with this model, the M_4_ PAM VU0152100 attenuated the D_1_ agonist SFK81297-stimulated SFK Y416 phosphorylation in the rat striatum (Mao and Wang, 2015). Coadministration of SKF81297 and scopolamine consistently induced a synergistic increase in striatal Y416 phosphorylation (Mao et al., 2018).

## Concluding remarks

Early studies have evaluated the role of mACh receptors in the regulation of SFKs in transfected cells or stable cell lines expressing a specific subtype of mACh receptors. It was found that the M_1_ subtype exhibits a profound influence over SFK activity. Pharmacological stimulation of M_1_ receptors activates SFKs as evidenced by an increase in autophosphorylation of SFKs at a conserved residue (Y416) in the activation loop of SFKs. Activated SFKs then serve as an essential element in forming a signaling pathway relaying M_1_ signals to various downstream effectors, including receptors, ion channels, enzymes, etc. Recent studies attempted to define the role of mACh receptors in neurons. Remarkably, M_4_ receptors seem to show an inhibitory role in regulating SFKs in striatal neurons. The M_4_ receptor also works in concert with the D_1_ receptor to control SFK activity in striatonigral output neurons coexpressing M_4_ and D_1_ receptors. M_4_/D_1_-regulated SFKs are able to link integrated signals from these receptors to glutamate receptors, thereby determining the excitability of these neurons in relation to synaptic transmission and plasticity.

While evidence has shown the existence of the linkage of mACh receptors to SFKs, precise signaling pathway(s) linking the receptor to the kinase are less clear. The cAMP-PKA pathway has been implicated in connecting M_4_ receptors to Fyn in striatal neurons. More studies are needed to confirm the role of the cAMP-PKA pathway in this event and to determine whether the M_4_-mediated regulation of Fyn occurs in M_4_-bearing striatonigral output neurons. In addition, the selectivity of SFK members subjected to the regulation by mACh receptors needs to be explored and characterized in neurons. Five among nine members of SFKs are known to be present in the brain, including Src, Fyn, Yes, Lyn, and Lck (Omri et al., 1996; Kalia et al., 2004; Bongiorno-Borbone et al., 2005). These SFK members are also distributed at synaptic sites (Kalia and Salter, 2003). Thus, they are thought to constitute a set of regulators essential for the modulation of synaptic transmission and plasticity. Evidence has already been shown to support the contribution of Src and Fyn in this regard (Mao et al., 2017; Matrone et al., 2020; Rajani et al., 2021). Future studies will target other members of SFKs to elucidate their individual contributions.

Ionotropic and metabotropic glutamate receptors have been identified to be biochemical substrates of Src/Fyn (Groveman et al., 2012; Mao and Wang, 2016b; Jin et al., 2017). SFKs bind to the intracellular domain of glutamate receptors and phosphorylate these receptors at specific residues to regulate trafficking, subcellular and subsynaptic distribution, and functions of modified receptors. Through a SFK-dependent pathway, mACh signals may modulate glutamate receptors. Indeed, the M_1_ agonist potentiated GluN2B-containing NMDA receptors in hippocampal CA1 neurons via Src (Ishibashi et al., 2014). The M_4_ PAM VU0152100 reduced the D_1_ agonist-stimulated GluN2B Y1472 phosphorylation in striatal neurons (Mao and Wang, 2015), indicating that the D_1_-regulated NMDA receptor phosphorylation is subject to the inhibitory modulation by M_4_ receptors. In addition to glutamate receptors, SFKs are shown to target other local synaptic proteins and coordinate their responses to changing synaptic input. Moreover, the mACh-SFK coupling in the hippocampus and striatum is thought to be critical for maintaining normal memory, cognitive behavior, mood, and movement. Dysfunction of this coupling is linked to pathogenesis of various neuropsychiatric and neurological illnesses (Ge et al., 2020; Guglietti et al., 2021; Rajani et al., 2021; Portugal et al., 2022; Wang et al., 2022).

## Author contributions

L-MM: Conceptualization, Formal Analysis, Writing – original draft. LY: Validation, Writing – review and editing. X-PC: Validation, Writing – review and editing. JW: Conceptualization, Funding acquisition, Supervision, Validation, Writing – original draft.
